# Mechanisms underpinning the effect of exercise on the non-alcoholic fatty liver disease: review

**DOI:** 10.17179/excli2024-7718

**Published:** 2025-02-11

**Authors:** Mohamed Bekheit, Blessed Kamera, Laura Colacino, Anne Dropmann, Mirela Delibegovic, Fatema Almadhoob, Nemany Hanafy, Giovanna Bermano, Seddik Hammad

**Affiliations:** 1 Department of Surgery, NHS Grampian, Foresterhill Health Campus, Ashgrove Road, AB252ZN Aberdeen, UK; 2 Institute of Medical Sciences, Medical School, Foresterhill Health Campus, Ashgrove Road, AB252ZN Aberdeen, UK; 3 Molecular Hepatology Section, Department of Medicine II, Medical Faculty Mannheim, Heidelberg University, Theodor-Kutzer-Ufer 1-3, 68167, Mannheim, Germany; 4 St. Helens and Knowsley Teaching Hospitals NHS Trust, Prescot, Prescot, UK; 5 Group of Bionanotechnology and Molecular Cell Biology, Nanomedicine Department, Institute of Nanoscience and Nanotechnology, Kafrelsheikh University, 33516 Kafrelsheikh, Egypt; 6 Centre for Obesity Research and Education (CORE), School of Pharmacy and Life Sciences, Robert Gordon University, Sir Ian Wood Building, Garthdee Road, Aberdeen AB10 7GJ, UK; 7 Department of Forensic Medicine and Veterinary Toxicology, Faculty of Veterinary Medicine, South Valley University, 83523 Qena, Egypt

**Keywords:** non-alcoholic, fatty liver disease, metabolic liver disease, exercise, lifestyle

## Abstract

Non-alcoholic Fatty Liver Disease (NAFLD) - whose terminology was recently replaced by metabolic liver disease (MAFLD) - is an accumulation of triglycerides in the liver of >5 % of its weight. Epidemiological studies indicated an association between NAFLD and reduced physical activity. In addition, exercise has been shown to improve NAFLD independently of weight loss. In this paper, we aim to systematically review molecular changes in sedentary experimental NAFLD models vs. those subjected to exercise. We utilized the Preferred Reporting Items for Systematic Reviews and Meta-Analyses (PRISMA) checklist and standard review techniques. Studies were considered for inclusion if they addressed the primary question: the mechanisms by which exercise influenced NAFLD. This review summarized experimental evidence of improvements in NAFLD with exercise in the absence of weight loss. The pathways involved appeared to have AMPK as a common denominator.

See also the graphical abstract(Fig. 1).

## Introduction

### NAFLD definition, epidemiology, and pathogenesis

Non-alcoholic Fatty Liver Disease (NAFLD) - whose terminology was recently replaced by metabolic dysfunction-associated fatty liver disease (MAFLD) (El-Kassas and Alswat, 2025[33]) - is an accumulation of triglycerides in the liver of >5 % of its weight (Browning et al., 2004[16]; EASL et al., 2016[32]); NAFLD is frequently associated with metabolic syndrome (Chalasani et al., 2012[24]). The association with one of five metabolic features is a requirement for the diagnosis, otherwise, steatotic liver disease (SLD) is identified (El-Kassas and Alswat, 2025[33]). 

Epidemiological studies indicated an association between NAFLD and a sedentary lifestyle. Reduced physical activity to a day per week or less is associated with an increased probability of NAFLD (Perseghin et al., 2007[119]; Rector and Thyfault 2011[124]) and deranged liver enzymes (Lawlor et al., 2005[80]). Data from the Nonalcoholic Steatohepatitis Clinical Research Network (NASH-CRN) indicated that individuals with NAFLD (biopsy-proven) not achieving ≥75 min/week of vigorous exercise had higher odds of developing Non-Alcoholic Steatohepatitis (NASH), and if not exceeding 150 min/week their likelihood of developing advanced fibrosis increases (Kistler et al., 2011[75]). Similar findings were observed in adolescents (Nader et al., 2008[104]).

NAFLD is viewed as a silent pandemic in the West and the East (Farrell et al., 2013[36]; Lazarus et al., 2020[81]). The prevalence of NAFLD is estimated at 25 % of the global population with an overall mortality of 11.77 per 1000 person-years (Younossi et al., 2016[169], Younossi et al., 2018[168]). With an estimated prevalence of over a billion subjects affected, even among lean children, approximately 10 % (Nobili and Pinzani 2010[108]; Schwimmer et al., 2006[131]), the disease is labeled a pandemic (Loomba and Sanyal, 2013[91]).

The spectrum of NAFLD encompasses simple accumulation of fat to associated steatohepatitis and cirrhosis (Tiniakos et al., 2010[148]). NAFLD is associated with extensive health-related complications (Tomeno et al., 2020[150]). Among those complications is hepatocellular carcinoma (HCC) (Huang et al., 2021[63]; Nwosu et al., 2017[109]), which is the third leading cause of cancer-related deaths worldwide (Bertuccio et al., 2017[12]) with poor survival (Jemal et al., 2017[67]). The incidence of NAFLD-related HCC is likely to rise by 122 % in the USA in the next decade (Huang et al., 2021[63]) and the prevalence of NASH is projected to rise to 56 % by 2030 (Estes et al., 2018[34]). 

Previously the two-hit theory was prevalent and central to the progression of the disease, however, this theory is considered outdated (Alonso et al., 2017[6]; Friedman et al., 2018[39]). The inability of the liver to handle the primary metabolic substrates leading to the accumulation of toxic lipids is believed to be the central mechanism of the pathogenesis (Neuschwander-Tetri, 2010[105]) as described in different etiology-based diseases i.e. cholestasis (Dropmann et al., 2020[31]; Hammad et al., 2018[53]).

### Role of diet 

Dietary contents are believed to influence the development of NAFLD. Saturated fatty acids (SFA), simple sugars, and animal proteins modulate the triglyceride and antioxidant accumulation in the liver resulting in its damage (Berna and Romero-Gomez 2020[11]). SFA impairs glutathione-mediated balance in the oxidative stress mechanisms leading to the progression of NAFLD (Franko et al., 2018[37]). Monosaturated fatty acids and plant-based proteins are thought to protect the liver against harmful fatty deposition (Perdomo et al., 2019[116]). The consumption of unsaturated fatty acids appears to reduce denovo hepatic lipogenesis (Rosqvist et al., 2019[128]). Dietary approaches including Mediterranean, low fat and low carbohydrate could be beneficial in reversing hepatic steatosis (Parry and Hodson 2020[113]). Several guidelines integrate dietary measures against NALFD (Chalasani et al., 2012[23]; EASL et al., 2016[32]). 

### Role of exercise and lifestyle

Lack of physical exercise was observed to be associated with NAFLD (Perseghin et al., 2007[119]). The threshold was assessed in observational studies that indicated exercise frequency less than once a week is associated with NALFD as opposed to >3 times/week (Hsieh et al., 1998[61]). A variety of exercise thresholds and types were assessed (Zelber-Sagi et al., 2008[171]). Individuals with biopsy-proven NAFLD not performing vigorous physical activity ≥75 min/week were more prone to NASH or advanced fibrosis if not exceeding 150 min/week (Kistler et al., 2011[75]). Short-term physical inactivity increases body fat mass (Kump and Booth, 2005[77]) and reduces insulin sensitivity (Kump and Booth 2005[76]) and net lipogenesis (Blanc et al., 2000[13]). The lack of physical exercise is linked to increased intra-abdominal and hepatic adiposity (Hannukainen et al., 2007[55]), which is contrary to what is observed in individuals with habitual exercise (Iozzo et al., 2004[66]). Cessation of routine exercise affects fat-related multi-mechanisms implicated in the development of NAFLD (Rector et al., 2008[125]).

### NAFLD is reversible

Reversal of NAFLD has demonstratable health benefits (Perry et al., 2013[118]). However, current NAFLD treatments have a range of effectiveness of which, each potential treatment is associated with a different risk-benefit profile. Physical exercise, aerobic or resistance, also improves hepatic steatosis (Vilar-Gomez et al., 2015[153]). In the absence of weight loss, exercise reduced hepatic fat content by 40 % (van der Heijden et al., 2010[151]).

### The available strategies in practice

These lifestyle interventions, although efficacious in treating NAFLD, require high and sustained patient engagement. Therefore, in the real world, 239 of 261 patients failed to achieve the 7 % weight loss required for significant impacts on NAFLD progression (St George et al., 2009[140]). Even with face-to-face individually tailored educational and counselling sessions, patients still found it difficult to implement and sustain behavior changes to achieve a modest 2 % weight loss (Lazo et al., 2010[82]). The Look Ahead multicenter clinical trial observed an 8 % weight loss and 50 % reduction in hepatic steatosis in patients receiving weekly intensive support and group counseling sessions, whilst 26 % in the group that only had 3 sessions in 12 months, had new onset NAFLD compared to baseline (Takahashi et al., 2015[144]). Because of the very high failure rates of the above approaches, Bariatric surgery proved to improve the metabolic profile in the liver even before the weight change happens (Laursen et al., 2019[79]; Madsbad et al., 2014[92]). This indicates that mechanisms may not be directly linked to weight changes (Yang et al., 2016[165]). However, although effective, it is not risk-free (Campos et al., 2020[19]).

Potential pharmacotherapies, none of which is licensed for this specific clinical use, including antioxidants (Vitamin E, herbal and natural medicines), insulin sensitizers (Thiazolidinediones, Angiotensin Converting Enzyme (ACE)), lipid-lowering drugs, probiotics and symbiotics were tried (Alkhouri, 2020[3]; Attia et al., 2021[7]; Dibba et al., 2018[29]). Novel molecular targets including intestinal (Farnesoid X receptor) FXR, (Peroxisome proliferator-activated receptor), PPAR alpha agonists, anti-apoptosis agents, and thyroid hormone receptors are being assessed in clinical trials (Attia et al., 2021[7]). Exercise is particularly interesting as it improves NAFLD independently of weight loss (Attia et al., 2021[7]) and improves mental (Sharma et al., 2006[133]), cardiovascular health and multiple other health benefits (Pinckard et al., 2019[122]). 

With poor compliance, a pharmaceutical substitute may be appealing. However, the potential therapeutic targets are not well summarized in the literature. To shed light on the mechanism(s) behind these desirable effects of exercise in the context of NAFLD, we aim to systematically review molecular changes in sedentary experimental NAFLD models vs. those subjected to exercise. This review aims to summarize the critical molecular mechanisms underpinning the role of exercise in NAFLD. 

**Figure 2:**
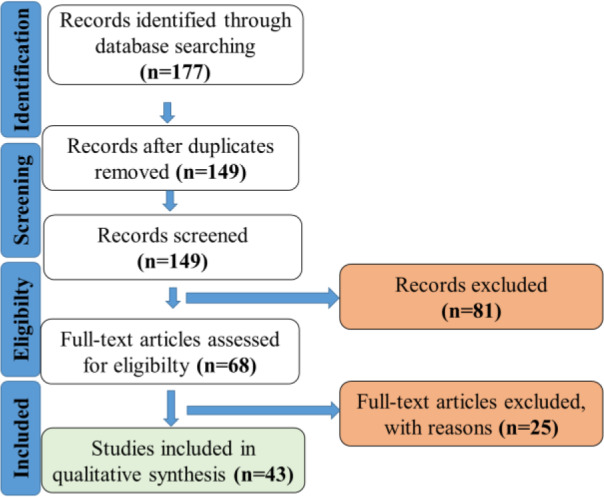
PRISMA chart outlining literature search strategy. The PRISMA chart shows the selection process followed to obtain the studies incorporated into the review. Titles and abstracts of 177 records were screened and 69 abstracts were selected for full-text review, of which 7 were only available as abstracts. Two papers were only available in Japanese and German. Sixteen full-text papers were excluded not meeting the inclusion criteria for one or more reasons, leaving 43 as the sources used for data analysis.

Towards that end, this review utilized the PRISMA checklist and standard review techniques (Dibba et al., 2018[29]) providing a systematic approach to the literature search ensuring the comprehensiveness and reproducibility of the results. Studies were considered for inclusion if they addressed the primary question: the mechanisms by which exercise influenced NAFLD. The review question was formulated according to the PICO structure. The population included experimental animals. The intervention focused on exercise, of any type. There was no specific comparison required for studies to be included in this review. Therefore, statistical summaries were not planned for this review. The primary outcome of interest was the reversal of NAFLD by exercise with qualitative assessment of the relevant molecules. An electronic search using the major electronic (EMBASE, Medline, The Cochrane Library, Ovid, Pubmed, and Web of Science) databases, was conducted (Figure 2(Fig. 2)). 

Two independent reviewers extracted 26 variables accounting for the type of animal model used to obtain NAFLD, find histological confirmation of features of NAFLD and reversal, and details of the exercise intervention used including duration and intensity were extracted. The study's primary endpoint was underpinning mechanisms by which exercise influenced NAFLD.

### Induction of NAFLD/NASH 

All studies had histological confirmation of NAFLD or NASH. NAFLD was achieved using either a calorie-concentrated diet or genetic manipulation causing hyperphagia. A variety of high-energy diets employing either fat (60-71 % fat) including D12492 (90 % lard, 10 % Soybean oil), Lieber Carli diet, D12451 diet or fructose as the calorie medium was employed. For moderate NAFLD, one study used a lower fat proportion (22 % fat) over 6 weeks (Hajighasem et al., 2018[50]; Homeyer et al., 2018[59]). A handful of studies used animals with mutated satiety genes leading to hyperphagia and obesity while feeding the animals' standard chow (Evangelista et al., 2015[35]; Haczeyni et al., 2015[49]; Linden et al., 2014[87], 2015[88], 2016[89]; Martinez et al., 2018[96]; Rector et al., 2008[125], 2011[126]). Manipulated genes include those coding for Phosphatase and Tensin Homolog (PTEN), Cholecystokinin (CCK) receptor, Alms gene (foz/foz model) and the leptin receptor in the Zucker rats. Several studies combined genetic manipulation and obesogenic diets (Batatinha et al., 2017[10]; Haczeyni et al., 2015[49]). Most of the included studies used the NAFLD activity score to describe the induced liver disease. One study observed the development of NAFLD after cessation of exercise rather than starting with NAFLD (Rector et al., 2008[125]). Forty-three studies formed the core findings of this review on the mechanisms underpinning the exercise-mediated NAFLD reversal/improvement.

### Animal characteristics

All studies employed rats or mice as the model of choice. The mouse model most frequently used was the male C57BL/6J used in 14 studies (Alex et al., 2015[2]; Batatinha et al., 2017[10]; Carbajo-Pescador et al., 2019[21]; Evangelista et al., 2015[35]; Ghareghani et al., 2018[41]; Goncalves et al., 2014[42], 2016[43]; Homeyer et al., 2018[59]; Kawanishi et al., 2012[71]; la Fuente et al., 2019[78]; Manta et al., 2022[94]; Marques et al., 2010[95]; Munoz et al., 2018[103]; Rector et al., 2011[126]). The Sprague Dawley and Wistar rats were also used (Goncalves et al., 2014[44], 2015[45]; Guo et al., 2020[47]; Martinez et al., 2018[96]; Passos et al., 2015[114]). One study used male Swiss mouse models instead (Piguet et al., 2015[121]).

To study the molecular mechanism of the exercise effect, some studies employed strategic mutations. Tang et al., used Macrophage Migration Inhibitory Factor knockout (MIF KO) mice (Tang et al., 2019[146]) while Batatinha et al., used PPAR alpha KO models (peroxisome-proliferator-activated receptor alpha) (Batatinha et al., 2017[10]). Winn and co-workers were employing estrogen alpha (ER alpha) knockouts to study the role of MIF, PPAR alpha and estrogen alpha receptors (Winn et al., 2019[159]). All but two studies used male animals (Batatinha et al., 2017[10]; Rector et al., 2008[125]) ranging in age from 3 weeks to 50 weeks. 

The animal protocols were similar in most studies, with the environment kept between 20 and 25 °C, 12 hr light/dark cycles and humidity at 35-60 %. All the studies provided ad libitum access to food and water.

A few studies varied their design from the usual control vs. intervention setup. Two examples are Tan et al. (2018[145]) and Younan et al. (2019[167]), who utilized female animals but also subjected one group to ovariectomy in addition to the exercise intervention to simulate NAFLD post-menopause. In another study, the animals with NAFLD induced by a high-fat diet were switched back to a normal diet after 16 weeks to simulate dietary intervention (Cho et al., 2016[26]).

### Exercise interventions

The exercise interventions are summarized in Table 1(Tab. 1) (References in Table 1: Alex et al., 2015[2]; Batatinha et al., 2017[10]; Borges Canha et al., 2017[14]; Botezelli et al., 2010[15]; Cameron et al., 2012[18]; Carbajo-Pescador et al., 2019[21]; Cho et al., 2016[26]; Evangelista et al., 2015[35]; Frantz et al., 2017[38]; Gehrke et al., 2019[40]; Ghareghani et al., 2018[41]; Goncalves et al., 2014[42], 2014[44]; Guo et al., 2020[47]; Haczeyni et al., 2015[49]; Hajighasem et al., 2018[50]; Henkel et al., 2019[58]; Hu et al., 2013[62]; Huber et al., 2017[64]; Kapravelou et al., 2015[70]; Kawanishi et al., 2012[71]; Khaleghzadeh et al., 2020[72]; Khoo et al., 2020[73]; la Fuente et al., 2019[78]; Lima et al., 2016[86]; Linden et al., 2014[87], 2015[88], 2016[89]; Marques et al., 2010[95]; Martinez et al., 2018[96]; Munoz et al., 2018[103]; Ok et al., 2018[111]; Passos et al., 2015[114]; Pereira et al., 2019[117]; Piguet et al., 2015[121]; Rector et al., 2008[125]; Schultz et al., 2012[130]; Scott et al., 2004[132]; Silva et al., 2014[137]; Tan et al., 2018[145]; Tang et al., 2019[146]; Winn et al., 2019[159]; Wu et al., 2015[162]; Younan et al., 2019[167]; Yu et al., 2019[170]) and were mostly motorized treadmill running but also included wheel running, swimming, and strength training utilizing both weights and aerobic exercise. 

Treadmill and voluntary wheel running were used in 23 (53.5 %) (Alex et al., 2015[2]; Batatinha et al., 2017[10]; Borges Canha et al., 2017[14]; Cameron et al., 2012[18]; Carbajo-Pescador et al., 2019[21]; Evangelista et al., 2015[35]; Frantz et al., 2017[38]; Ghareghani et al., 2018[41]; Goncalves et al., 2016[43]; Guo et al., 2020[47]; Henkel et al., 2019[58]; Hu et al., 2013[62]; Kawanishi et al., 2012[71]; Khaleghzadeh et al., 2020[72]; la Fuente et al., 2019[78]; Lima et al., 2016[86]; Linden et al., 2014[87], 2015[88]; Marques et al., 2010[95]; Martinez et al., 2018[96]; Munoz et al., 2018[103]; Passos et al., 2015[114]; Winn et al., 2019[159]) and 10 (23.3 %) (Gehrke et al., 2019[40]; Goncalves et al., 2015[45], 2016[43]; Haczeyni et al., 2015[49]; Huber et al., 2017[64]; Khoo et al., 2020[73]; Passos et al., 2015[114]; Rector et al., 2011[126]; Winn et al., 2019[159]; Yu et al., 2019[170]) of the studies; respectively. Only 3 of the voluntary wheel running (VWR) reported the range of distances run (Dibba et al., 2018[29]; Hu et al., 2013[62]; Munoz et al., 2018[103]). Some of the treadmill running studies set the training at 50-75 % of the predetermined maximum speed (Batatinha et al., 2017[10]; Linden et al., 2014[87]; Martinez et al., 2018[96]; Rodrigues Prestes et al., 2017[127]; Schultz et al., 2012[130]; Tang et al., 2019[146]). Five studies used lane swimming while one used a barrel as their chosen mode of exercise (Rodrigues Prestes et al., 2017[127]; Schultz et al., 2012[130]; Tang et al., 2019[146]; Wu et al., 2015[162]; Younan et al., 2019[167]). Two studies added a weight attached to the animals as they swam to increase the work of training (Botezelli et al., 2010[15]; Silva et al., 2014[137]). One study loaded the knee with 1 N at 10 Hz for 6 min per day, 5 days per week for 6 weeks (Tan et al., 2018[145]). Also bucking the trend were Pereira et al. who utilized a form of strength training (carrying weights up a ladder) (Pereira et al., 2019[117]). Rector and co-workers employed a different strategy by forcing sedentariness on one group of animals vs. those at liberty to exercise (Rector et al., 2008[125]). Some studies also reflected the growing trend in high-intensity interval training (HIIT) and employed short but vigorous aerobic exercise (Khaleghzadeh et al., 2020[72]; Linden et al., 2015[88]; Martinez et al., 2018[96]). Two of these studies were treadmill running at 80-95 % of the predetermined maximum capacity (Khaleghzadeh et al., 2020[72]; Linden et al., 2015[88]). Martinez et al. combined moderate running and aerobic strength training to provide 60 mins of HIIT (Martinez et al., 2018[96]). Importantly, some studies compared the effectiveness of different forms of exercise in attenuating NAFLD. Moderate treadmill running was compared with HIIT and endurance running (Goncalves et al., 2016[43]). Voluntary wheel running vs. endurance training was reported by Goncalves and co-workers (Goncalves et al., 2014[42], 2014[44], 2016[43]). Finally, some studies acknowledged an improvement in peak performance by re-evaluating maximum performance part way through the experiment and re-pegged the level of exercise intensity.

## Molecular Changes in NAFLD/Exercise vs. NAFLD/Sedentary

### Lipid metabolism

The 1^st^ stage of NAFLD, simple steatosis, results from an impairment in the four main processes that affect lipid metabolism homeostasis: de novo lipogenesis, fatty acid uptake from circulation, fatty acid oxidation and lipid secretion from the liver as very high-density lipoproteins (Hammad et al., 2017[52], 2023[54]; Mungamuri et al., 2021[102]). Several studies found no difference in the transcription factors and enzymes involved in de novo lipogenesis when comparing NAFLD/sedentary vs. NAFLD/exercise groups (Gehrke et al., 2019[40]; Khoo et al., 2020[73]; Ok et al., 2018[111]; Piguet et al., 2015[121]). Others found an increase in sterol regulatory element binding transcription factor (Srebp1c, a NAD dependent deacetylase), the main transcription factor for proteins involved in synthesizing fatty acids, fatty acid synthase (FAS), and Acetyl Co-A carboxylase in the NAFLD/sedentary group (Carbajo-Pescador et al., 2019[21]; Ghareghani et al., 2018[41]; Guo et al., 2020[47]; Linden et al., 2014[87], 2015[88]; Martinez et al., 2018[96]; Munoz et al., 2018[103]; Pereira et al., 2019[117]; Piguet et al., 2015[121]; Rector et al., 2011[126]). However, one study attenuated FAS in the NAFLD/exercise group by 60 % (Schultz et al., 2012[130]). Carnitine palmitoyl-transferase 1 (CPT1a) - rate-limiting enzyme for fatty acid oxidation - increased in the NAFLD/exercise more than in the NAFLD/sedentary groups (Almeida et al., 2013[5]; Peng et al., 2011[115]; Wu et al., 2015[162]). Other studies found a conflicting trend i.e. attenuated CPT1a levels in the NAFLD/sedentary but increased in the NAFLD/exercise groups (Evangelista et al., 2015[35]; Ok et al., 2018[111]; Pereira et al., 2019[117]). Two studies observed a decrease in the translation and protein expression of the main lipid uptake transporter Cluster-of-differentiation-36 (CD36)/ Fatty-Acid-translocase (FAT) following exercise of the NAFLD models (Carbajo-Pescador et al., 2019[21]; Linden et al., 2016[89]). The other lipid uptake transporter, Fatty Acid Binding Protein (FABP1) was increased in NAFLD and attenuated by exercise, while its over-expression reversed all exercise effects (Pi et al., 2019[120]).

### Mitochondrial bioenergetics

Hepatocyte mitochondria play a pivotal role in the homeostasis of lipid metabolism by facilitating fatty acid (FA) oxidation (Middleton and Vergis, 2021[98]). Increased FA oxidation causes increased production of reactive oxygen species (ROS), which increases oxidative stress, damages DNA and organelles, and impairs mitochondrial function; this manifests as dysfunction of the electron transport chain with uncoupling of proton movements from ATP production. Reduced mitophagy leading to increased hepatocyte apoptosis is also associated with mitochondrial dysfunction.

The genes coding for molecules involved in mitochondrial bioenergetics, namely mitochondrial transcription factor A (Tfam), peroxisome proliferator-activated receptor alpha (PGC1-alpha), nuclear respiratory factor 1 (NRF1), mitochondrial transcription factors Tfb1m and Tfb2m, had different expression between the NAFLD/exercise vs NAFLD/ sedentary (Khoo et al., 2020[73]). Meanwhile, others found an increase in translation of Tfam and PGC-alpha in the NAFLD model, which was increased by exercise (Ghareghani et al., 2018[41]; Wang et al., 2017[154]). In another study, only Tfam was reduced in the NAFLD/sedentary models while both PGC 1alpha and Tfam were increased by exercise (Goncalves et al., 2016[43]). Two studies found an increased number of abnormal mitochondria, loss of cristae, increased susceptibility to calcium-induced mitochondrial swelling, and reduced intramitochondrial granules in the NAFLD/sedentary animals, but this was reduced by exercise (Goncalves et al., 2014[42], 2016[43]). The electron transport chain was impaired in the NAFLD/ sedentary models including phospholipid composition of the mitochondrial membrane, favoring a less fluid combination with less integrity, less membrane potential, more susceptibility to respiratory uncoupling, and reduced oxidative phosphorylation capacity (Goncalves et al., 2014[44], 2016[43]). At least, the increase in ATP production was more pronounced in NAFLD/ exercise compared to NAFLD/sedentary (Peng et al., 2011[115]).

### Autophagy markers - changes with exercise and the development and progression to NAFLD

The role of autophagy in NAFLD is contextual, promoting lipolysis in the fed state and lipogenesis in the nutrient-deficient state (Niture et al., 2021[106]). In diet-related NAFLD, autophagy promotes mitochondrial beta-oxidation and degradation of lipids and regulates their storage in hepatocytes (Wang et al., 2019[155]). Downregulation/knockout of the autophagy apparatus such as Atg5, Atg3 (autophagy-related-5 and 3), Beclin 1 and LC3 (Microtubule-associated protein 1A/1B-light-chain-3) lead to impaired autophagy, and incomplete mitochondrial beta-oxidation resulting in the accumulation of hepatic triglycerides and toxic lipid intermediates, causing mitochondria-induced apoptosis (Hammad et al., 2023[54]; Pi et al., 2019[120]; Wang et al., 2019[155]). Steatotic hepatocytes have attenuated levels of autosomal acidification, Cathepsin-B, L-protease, and accumulation of autophagosomes, demonstrating impaired enzymatic autolysis (Niture et al., 2021[106]). We observed Atg5, Atg7, LC3, and Beclin 1 to be attenuated in the NAFLD model but increased in the NAFLD/exercise group (Ghareghani et al., 2018[41]; Tan et al., 2018[145]; Wang et al., 2017[154], 2019[155]). In some studies, mixed results were obtained (Pi et al., 2019[120]; Wang et al., 2017[154]). The mammalian target of Rapamycin-1 (mTOR1) is a serine/threonine kinase which negatively regulates autophagy. It phosphorylates and inactivates three key elements of phagosome formation, ULK1 (Unc-51-like autophagy-activating-kinase-1), Atg14 (autophagy-related-protein), and AMBRA1 (activating molecule in Beclin1-regulated-autophagy-protein-1) and modifies the transcription of genes related to lysosome function (Wang et al., 2019[155]). In this review, exercise attenuated mTOR1 expression, which improved autophagy (Ghareghani et al., 2018[41]; Piguet et al., 2015[121]; Wang et al., 2017[154]). This was associated with a rise in AMPK (AMP-activated protein-kinase) levels (Ghareghani et al., 2018[41]). MicroRNA 33 (miR33) was studied by Ghareghani et al., who observed an attenuated miR33 expression in the NAFLD/sedentary mice, nearly doubled by exercise training p<0.05 (Ghareghani et al., 2018[41]; Kim et al., 2021[74]). Using a miR33 mimic, mRNA expression of Beclin-1 was induced by 184 %, while Atg5, Atg7, and LC3 were increased by 56 %, 62 %, and 48 % respectively, showing that miR33 positively regulates autophagy (Ghareghani et al., 2018[41]).

## Mechanistic Theories for Exercise-Induced Reversal of NAFLD

Among the studies selected, the pathways that were most often found to have a role in NAFLD/NASH and were affected by exercise were those related to mitochondrial biosynthesis and energy homeostasis, apoptosis, inflammation, lipogenesis, and insulin release. The following list highlights the most commonly affected molecules and the pathways in which they work.

### AMP-activated protein kinase (AMPK)

Exercise is a potent activator of AMPK through phosphorylation, which decreases intrahepatic de novo lipogenesis and intrahepatic triacylglycerol accumulation (Bae-Gartz et al., 2020[9]; Batatinha et al., 2017[10]; Ghareghani et al., 2018[41]; Ok et al., 2018[111]). AMPK, a key regulator of cellular homeostasis, has a strong role in fatty acid oxidation and intrahepatic lipogenesis. AMPK was also found to be more abundant in its phosphorylated state in the livers of lean compared to obese mice (Bae-Gartz et al., 2020[9]). Increased AMPK-alpha activity following exercise inhibited lipogenesis and reduced hepatic fatty acid B-oxidation (Gehrke et al., 2019[40]; Ok et al., 2018[111]). AMPK is essential in upregulating genes related to mitochondrial biosynthesis and oxidative enzymes and is associated with lipid oxidation, especially with Macrophage migratory inhibitory factor, which is an activator of AMPK (Moon et al., 2013[100]).

### Akt strain transforming (AKT)

Akt is involved in the protein-kinase-B (Akt)/phosphatidylinositol-3-kinase (PI3K)/ insulin receptor substrate (IRS-1) insulin signaling pathway, which improves hepatic insulin resistance and therefore causes the ACE2/Ang(1-7)/Mas axis to increase glucose uptake, enhance glucose tolerance and insulin sensitivity, decrease glycogen synthesis and reduce stress in hepatic cells (Wang et al., 2024[157]). Exercise was crucial in restoring this axis to normal levels compared to untrained rats (Frantz et al., 2017[38]; Munoz et al., 2018[103]; Pereira et al., 2019[117]). Exercise also enhanced phosphorylation of the serine kinase Akt (Gehrke et al., 2019[40]). Akt is upstream of mTOR - linked to induction of autophagy, found to have diminished levels during exercise (Ghareghani et al., 2018[41]). Autophagy is implicated in the amelioration of NASH when stimulated through chronic exercise, and p-AKT (phosphorylated Akt) may participate in other signaling pathways such as apoptosis, interacting with other pro-apoptotic molecules (Guo et al., 2020[47]).

### Sirtuin 1 (SIRT1)

SIRT1 increases with exercise and is an NAD-dependant deacetylase that protects against NAFLD by inhibiting NF-κB and CD36 thus decreasing hepatic inflammation and steatosis (Niu et al., 2018[107]).

### Macrophage inhibitory factor (MIF)

MIF is hepatoprotective in a CD74/ AMPK mediated pathway and is a cytokine expressed by immune cells, hepatocytes and Kupffer cells resulting in a reduction of triglyceride content and hepatic inflammation. MIF is involved in regulating the Akt pathway, is downregulated in patients with NAFLD and high-fat diets, and is induced by exercise (Tang et al., 2019[146]).

### MicroRNA33 (MiR33)

MiR33, a non-coding RNA found in the intron of SREBP1c was reduced in the NAFLD while the lipogenic transcription factor SREBP1c and its target gene increased. The reverse was seen in the NAFLD/exercise animals where miR33 was restored while mRNA and protein levels of SREBP1, FAS and ACC were reduced (Ghareghani et al., 2018[41]).

### Adiponectin

Adiponectin activates hepatic AMPK and ACC (Acetyl-CoA-carboxylase). ACC catalyses the carboxylation of Acetyl-coA to produce malonyl-CoA which then feeds into the lipogenesis pathway. AMPK phosphorylates ACC to inactivate it. In this study, serum and adipocyte adiponectin levels were reduced in the NAFLD model (Gehrke et al., 2019[40]). Hepatic AdipoR1 and R2 receptors were downregulated at both mRNA and protein levels, with effects reversed by exercise.

### Liver-X receptor (LXR) and Farnesoid-X Receptor (FXR)

LXR is an oxysterol-activated nuclear receptor responsible for the regulation of major metabolic pathways for cholesterol homeostasis, bile acid metabolism and lipogenesis (Ahn et al., 2014[1]). FXR is a ligand-activated transcription factor abundantly expressed in the liver. Its hepatoprotective role is essential for normal liver function, it is critical in regulating lipid metabolism and suppressing hepatic inflammation (Zhu et al., 2016[174]). LXR and FXR gene expression of Elderly Wistar rats on an HFD (Hajighasem et al., 2018[50]) was measured and normalized to show a significant increase in LXR and FXR in exercised vs. sedentary animals (p<0.01), associated with a decrease in liver enzymes, triglycerides (p<0.0000) and hepatocyte apoptosis (p<0.0001) (Hajighasem et al., 2018[50]). 

### Estrogen alpha receptors (ER-α)

ER-α receptor's binding with AMPK is critical in the pathogenesis of NAFLD (Lipovka et al., 2015[90]). In ER-α knockout mice, HFD induced NAFLD and in wildtype animals, there was an increase in muscle but not liver ER-α protein expression (Winn et al., 2019[159]). The absent ER-α in the KO model was associated with 30 % greater hepatic triglycerides vs. the wildtype, not improved by exercise. Exercise in the wild-type animals halved hepatic triglycerides. Similarly, inflammatory markers F4/80 and CD68 were elevated in the KO animals vs the wildtype attenuated by exercise (Winn et al., 2019[159]). Loss of ER-α signaling leads to hepatic inflammation, increased insulin resistance and glucose intolerance leading to NAFLD (Winn et al., 2019[159]).

### Renin-angiotensin system

The renin-angiotensin pathway plays a role in the development of inflammation and fibrosis in the liver (Velloso et al., 2006[152]). It is the ACE2-Ang(1-7)-MAS axis of the pathway, via the MAS receptor, which demonstrated in experimental studies to reduce inflammation, proliferation, and fibrosis via attenuated release of cytokines (Rodrigues Prestes et al., 2017[127]). Inhibition of ACE in experimental NAFLD models led to improvement in NAFLD (Frantz et al., 2017[38]). Ca et al. observed that Ang(1-7) reduces the production of reactive oxygen species (ROS) in hepatocytes (Cao et al., 2016[20]). One study observed the highest ACE protein expression in the NAFLD/Sedentary animals > NAFLD/exercise > Control/sedentary > control/exercise (p<0.05). In addition, liver ACE activity and liver angiotensin 2 increased in the sedentary/NAFLD model vs the control/exercise animals to 199.6 % and 164 %, respectively (p<0.05). There was no significant difference in the Angiotensin-1 receptor protein levels between sedentary and exercised NAFLD animals. Liver angiotensin1-7 showed an opposite trend, increasing threefold in the NAFLD/exercise group vs the NAFLD/sedentary, P<0.05. Liver ACE2 was not significantly different between the groups. Expression of MAS receptor which binds angiotensin (1-7) was lower in the NAFLD/sedentary group than in the exercise groups (p< 0.05) (Frantz et al., 2017[38]).

### Hydrogen sulphide (H_2_S)

H_2_S is a gastrotransmitter, increasingly recognized as having an important role in the pathogenesis of liver diseases and cirrhosis. H_2_S metabolism has important effects on glucose metabolism, insulin sensitivity, lipoprotein synthesis and mitochondrial processes (Mani et al., 2014[93]). In the NAFLD model vs control, H_2_S levels in plasma and liver, relative mRNA expression of cystathionine β-synthase (CBS), cystathionine γ-lyase (CES), and 3-mercaptopyruvate sulfotransferase (3-MST) in the liver showed a significant reduction. Chronic exercise caused significant enhancement in H_2_S levels in plasma and liver, and relative mRNA expression of CBS, CES, and 3-MST in livers of the NAFLD model (Henkel et al., 2019[58]).

### Activin/follistatin

The role of activin and follistatin in NAFLD is not well understood. Activin A is part of the transforming-growth-factor-B superfamily (TGF-B). Activins primarily signal through transmembrane serine/threonine kinase receptors to regulate the transcription of target genes involved in cell proliferation, differentiation, wound healing, apoptosis, and metabolism (Colucci et al., 2021[27]; McDowell et al., 1997[97]; Mohamed et al., 2024[99]). In hepatocytes, activin A and follistatin were beneficial against lipid accumulation but may promote hepatic inflammation and fibrosis (Yndestad et al., 2009[166]). Activin can inhibit hepatocyte replication, induce hepatocyte apoptosis and inhibit insulin sensitivity, attenuating glucose and lipid metabolism (Silva et al., 2014[137]). Conversely, follistatin is a glycoprotein that binds activin and can promote liver regeneration (Silva et al., 2014[137]). Silva et al. found that mRNA expression of activin A was highest in the NAFLD/exercise vs NAFLD/sedentary animals while the reverse was true for follistatin. Based on the posited downstream effects of activin A, one would expect post-transcriptional regulation (Silva et al., 2014[137]).

### Gut microbiota

Disruption of the gut microbiota plays a significant role in the pathogenesis of NAFLD. Two studies observed normal livers in germ-free animals on HFD which then developed NAFLD following fecal transplantation from NASH patients (Burz et al., 2021[17]; Chiu et al., 2017[25]). While no specific dysbiosis is consistently linked with NAFLD, there is a decrease in bacterial diversity with attenuated levels of Firmicutes and Clostridia and increased Bacteroidetes (Tokuhara, 2021[149]). The pattern of dysbiosis will vary with age, BMI and stage of progression in NAFLD. The mechanism by which the dysbiosis results in NAFLD is multifactorial and includes increased gut permeability, increased lipopolysaccharide influx and abnormal production of short fatty acids chains (Tokuhara, 2021[149]). One study made similar observations: exercise attenuated the gut barrier disruption induced by HFD, decreased the migration of LPS and reduced oxidative stress (Carbajo-Pescador et al., 2019[21]). Exercise caused the opposite gut microbiota profile to that induced by HFD with a higher Firmicutes: Bacteroidetes ratio similar to that of the control subjects, associated with an improvement in NAFLD (Carbajo-Pescador et al., 2019[21]).

### Hepatic CLK2 (CDC-like kinase-2)

CLK2 was identified as an insulin-responsive repressor of hepatic gluconeogenesis in the fed state via interactions with Akt and PGC1-alpha (Tabata et al., 2014[143]). In the fasted state, CLK2 levels were suppressed, while in the fed state they increased and attenuated hepatic triglyceride accumulation by phosphorylating PGC1-alpha, resulting in reduced gene transcription of proteins involved in fatty acid oxidation and ketogenesis. In this review, one study posited the theory that CLK2 modulation could be how exercise attenuates NAFLD. They found that the NAFLD/exercise animals showed a significant reduction in the hepatic CLK2 content compared to NAFLD/sedentary ones (Munoz et al., 2018[103]). An increased association between Akt and CLK2 in the NAFLD/sedentary group was attenuated in the NAFLD/exercise group. Exercise did not affect CLK2 and PGC1alpha interaction. 

### Fatty acid-binding protein

The role of fatty acid binding protein (FABP) in NAFLD is unclear. Transcription activators of FABP, PPAR alpha and FOXA1 (Fox-head Box protein A1) were reduced in NAFLD while its repressor CCAAT/enhancer-binding protein-alpha (C/EBPα) was induced or unchanged (Guzman et al., 2013[48]). Mukai and co-workers used FABP knock-out high-fat diet NAFLD mice models to show an attenuation of de novo lipogenesis, hepatic inflammation and oxidative stress (Mukai et al., 2017[101]). However, it has been observed a 1.8-fold increase in FABP in the HFD-induced C57BL/6J male mice model vs. control which was attenuated to 1.2-fold by exercise (Pi et al., 2019[120]; Song et al., 2020[139]).

## Exercise and NAFLD

Exercise is part of the proposed treatment for NALFD (Aller et al., 2018[4]). However, despite its effectiveness, the processes by which the exercise improves the NALFD are not clear (recently reviewed by Xue and co-workers, 2024[163]. Exercise appears to reverse many of the processes contributing to the development of NAFLD. In this review, pivotal molecules in de novo lipogenesis were shown to increase in the NAFLD model and consistently decrease with exercise. The transcription and translation of SREBP1c and PPAR alpha, both master transcription factors in the regulation of enzymes involved in lipogenesis including FAS, ACC, Elov6, SCD1, CD36/ FAT decreased and increased with exercise respectively (Carbajo-Pescador et al., 2019[21]; Evangelista et al., 2015[35]; Ghareghani et al., 2018[41]; Guo et al., 2020[47]; Linden et al., 2014[87], 2015[88]; Pereira et al., 2019[117]; Piguet et al., 2015[121]; Rector et al., 2011[126]; Schultz et al., 2012[130]).

Post-translation effects of exercise were also noted, such as increased phosphorylation of acetyl-co-A-carboxylase, deactivating it and reducing de novo lipogenesis. Inhibition of ACC decreased hepatic lipids by 36 % while ACC KO models were protected from NAFLD (Pereira et al., 2019[117]). The inaction of ACC leads to less inhibitory regulation of Cpt1a and more AcetylCoA shunting towards beta-oxidation which increased with exercise. Key players in beta-oxidation improved with exercise including citrate synthase and beta-HAD (Rector et al., 2011[126]). Exercise increased Cpt1a in NAFLD, the rate-limiting enzyme for beta-oxidation, from normal (Almeida et al., 2013[5]; Wang et al., 2017[154]) and reduced its baseline levels (Evangelista et al., 2015[35]; Ok et al., 2018[111]; Pereira et al., 2019[117]; Yan et al., 2018[164]). 

Cpt1a acts in the mitochondria, the structure and function of which were also affected by NAFLD and exercise. The many NAFLD-related impaired functions of mitochondria were protected or enhanced by exercise. Exercise increased the number of mitochondria and reduced loss of cristae, susceptibility to Ca2+-induced swelling and loss of intramitochondrial granules (Goncalves et al., 2014[42], 2014[44], 2015[45]). Both mitochondrial biogenesis and mitophagy increased with exercise, giving hepatocytes resilience to different stressors induced by NAFLD with increases in PGC1alpha, Tfam, Mfn1 and Mfn2 (Ghareghani et al., 2018[41]; Goncalves et al., 2014[42], 2014[44], 2015[45]; Khoo et al., 2020[73]; Wang et al., 2017[154]). Exercise influenced the composition of the mitochondrial membrane, favoring less uncouplers as well as increased membrane fluidity and integrity, preserving the RCR (Goncalves et al., 2014[44]). 

Exercise decreased oxidative stress, and ATP synthase, glutamate dehydrogenase and ACADL (long-chain-specific acyl-coenzyme-A) were protected from oxidation-induced damage seen in sedentary animals but absent in those exercising (Hu et al., 2013[62]). Markers of oxidative stress, like MDA, were reduced by exercise (Hu et al., 2013[62]; Wang et al., 2017[154]; Yu et al., 2019[170]), possibly due to upregulation of both transcription factors of the antioxidant system, such as Nrf2, and their downstream targets including superoxide dismutase (SOD), the glutathione system, thiols and catalase (Batatinha et al., 2017[10]; Guo et al., 2020[47]; Wang et al., 2017[154]; Yu et al., 2019[170]). A few results contradicted the above with a decrease in SOD, glutathione system components, catalase and thiols while MDA levels were unaffected following exercise (Hu et al., 2013[62]; Kapravelou et al., 2015[70]; Yu et al., 2019[170]). This could be explained partly by the difference in the applied model and exercise regimens. 

Enhanced hepatocyte apoptosis; one feature of NAFLD shown by high levels of the pro-apoptosis pathway such as a high BAX: BeCl2 and cleaved Caspase 3 which were reduced by exercise (Guo et al., 2020[47]). Caspases are activated in NAFLD by the release of Cytochrome C from the mitochondria into the cytosol, with the same outcome of apoptosis, however, exercise confined this process to the mitochondria causing controlled mitophagy (Guo et al., 2020[47]).

Hepatocyte autophagy, useful for recycling damaged or defective cell organelles, decreased in NAFLD. Exercise improved mitochondrial autophagy flux and reduced NAFLD that was caused by the damaged mitochondria leading to incomplete beta-oxidation, increased oxidative stress, accumulation of toxic lipid intermediates and increased mitochondria-mediated apoptosis (Wang et al., 2017[154]). Downregulation of markers and intermediates such as p62, LCIII, LAMP2 and Beclin 1 at both mRNA and protein levels was observed in the NAFLD model and reversed by exercise (Ghareghani et al., 2018[41]; Pi et al., 2019[120]; Tan et al., 2018[145]). Pi et al. demonstrated a reversal of the dysfunction of the lysosomal proteases affected by the NAFLD diet by exercise (Pi et al., 2019[120]).

## Translation to Human

Only a few studies examined the influence of exercise on NAFLD independently from the dietary modifications due to the lack of an optimal experimental model mimicking NAFLD in patients (Othman et al., 2020[112]; Teufel et al., 2016[147]). Devries and colleagues (2008[28]) and Shojaee-Moradie et al. (2007[135]) described no significant change in hepatic lipid content measured non-invasively CT-scan complemented with surrogate markers following 6 to 12 weeks of endurance exercise. On the other hand, Johnson and co-workers (2009[69]) and van der Heijden et al. (2010[151]) demonstrated a meaningful reduction in hepatic fat content measured by MRI spectroscopy following endurance exercise. This may also shed light on the relative sensitivity of the tools utilized for assessment (Starekova et al., 2021[141]). 

## Deducing the Mechanism Linking Exercise to its Effect on NAFLD

Several pathways were considered by the studies included in the review as the link between exercise and its effects on NAFLD. Song et al. deduced that the ACE2-(Ang1-7)-Mas axis, via Akt and AMPK, was a key pathway (Song et al., 2020[139]). MIF increased significantly after 4 weeks of exercise and was accompanied by an increase in AMPK phosphorylation along with its downstream effects (Moon et al., 2013[100]). *In vitro* addition of MIF to hepatocytes confirmed the increase in pAMPK while AMPK inhibitors cancelled the effects of MIF (Tang et al., 2019[146]). This suggests that MIF is protected against NAFLD via the AMPK pathway. The link between exercise and MIF could be due to its role as an energy sensor and induction by conditions that reduce ATP or hypoxia (Moon et al., 2013[100]). 

Independent of the circulating estrogen levels, the downregulation of ER-α increases steatosis and proinflammatory gene expression (Winn et al., 2019[159]). Ovariectomized mice showed more severe NAFLD compared to the controls (Tan et al., 2018[145]; Younan et al., 2019[167]). The metabolomics of these studies showed estrogen binding to ER as having a significant role in autophagy, fatty acid oxidation and TG export out of the liver, all of which were improved by exercise (Younan et al., 2019[167]). Lipovka et al. suggested direct binding of ER-α to the alpha subunit of AMPK may be the mechanism by which the ERs mediate their effect (Lipovka et al., 2015[90]).

Adiponectin was considered protective against NAFLD. It stimulates the activation of hepatic AMPK and ACC. Exercise boosted serum, adipocyte adiponectin, hepatic AdipoR1 and R2 receptors at mRNA and protein levels, which were downregulated in NAFLD (Gehrke et al., 2019[40]). One systematic review concluded that while the evidence for the increase in Adiponectin with exercise was inconsistent, it could be as high as 38 % (Simpson and Singh, 2008[138]).

A CDC2-like kinase (CLK2) binds to PGC1alpha and inhibits its effects on fatty acid oxidation and ketogenesis. Therefore, exercise-induced reduction of CLK2 leads to less suppression of PGC1alpha and reduced NAFLD (Munoz et al., 2018[103]). MicroRNA33 regulates the expression of genes by degrading the transcribed mRNA or by binding to them and preventing ribosomal translation. HFD is linked to overexpression of SREBP1, which was reduced in the NAFLD model but restored by exercise (Ghareghani et al., 2018[41]), as a direct result of reduced miR33 regulation (Horie et al., 2013[60]). Exercise increases Activin-A, a TGF-beta cytokine, levels (Silva et al., 2014[137]), which appear to reduce hepatic steatosis and promote hepatic fibrosis by increasing the activity of MMP, the laying down of collagen3 (Yndestad et al., 2009[166]). Activation of the AMPK inhibits the SERBP1 leading to amelioration of hepato-steatosis (Li et al., 2011[85]).

SIRT1 increased with exercise with associated improvement in NAFLD and its markers (Ghareghani et al., 2018[41]) via inhibition of NF-κB and CD36 (Niu et al., 2018[107]). One review concluded that SIRT1 is involved in CD36 and NFκB pathways regulating de novo lipogenesis, oxidative stress and stimulating fatty acid beta-oxidation (Ding et al., 2017[30]). Moreover, SIRT1 is required for AMPK activation (Price et al., 2012[123]).

Hydrogen sulphide (H_2_S) was decreased in the NAFLD but reversed by exercise (Wang et al., 2017[154]). Although the mechanism implicating exercise in this reversal is not clear, Exogenous H_2_S donors protected hepatocytes from fatty acid-mediated inflammation, as well as suppressed liver oxidative stress by activating the P13K/Akt/HO-1 signaling pathway (Wu et al., 2020[161]). There seems to be an interaction between the AMPK activation and the cytoprotective effect of the H_2_S (Wang et al., 2017[156]), albeit this interaction is not fully elucidated.

## AMPK: the Common Denominator

**Figure 3:**
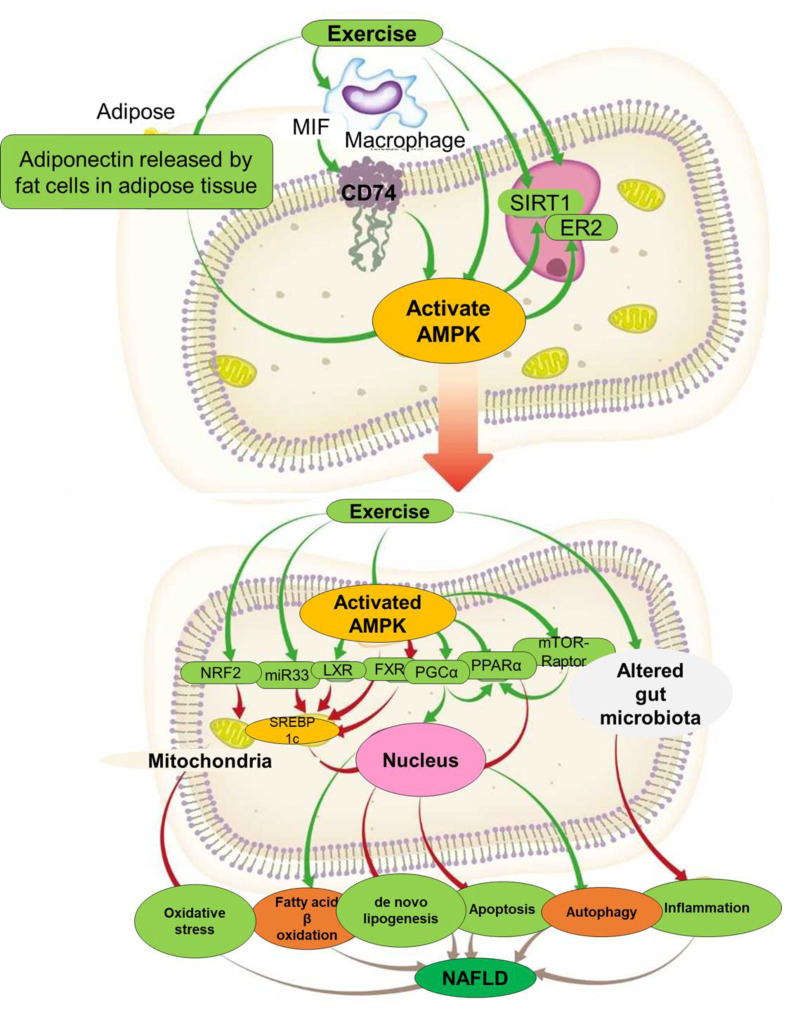
The suggested pathway from exercise to attenuated NAFLD appears to hinge on the kinase AMPK as a major pathway. The diagram illustrates the regulatory effects of exercise on adipose tissue, macrophages, and hepatocytes via the activation of AMPK (AMP-activated protein kinase) signaling pathways. Exercise stimulates adiponectin secretion from fat cells, which interacts with macrophages and receptors such as CD74 to influence SIRT1 and ER2 pathways. Activated AMPK orchestrates downstream signaling cascades, including nuclear transcription factors (NRF2, miR33, LXR, FXR, PGCα, PPARα), promoting beneficial metabolic effects. These effects include reduced oxidative stress, enhanced fatty acid β-oxidation, inhibited de novo lipogenesis, and improved autophagy. Collectively, these changes mitigate apoptosis and inflammation, contributing to the prevention or attenuation of NAFLD. Altered gut microbiota further modulates the AMPK pathway, highlighting the systemic interplay between different organs.

The AMPK appears to traffic several pathways/molecules linking exercise to its effects on NAFLD. The complex structure and function of AMPK lend itself to being a major link between exercise and most of the mechanisms by which it reverses NAFLD (Figure 3(Fig. 3)).

In 1989 the 3 subunits of AMPK were determined, with the alpha having the catalytic activity (Carling et al., 1989[22]; Hardie et al., 2016[56]). There are at least 2 isoforms of each subunit, giving 12 possible permutations whose effect on AMPK activity is unknown (Hardie et al., 2016[56]; Ross et al., 2016[129]). Activation of AMPK is by phosphorylation by upstream kinases of a threonine residue within the activation loop of the alpha-subunit kinase domain (Hardie et al., 2016[56]). 

Its gamma subunit contains 4 regulatory binding sites which bind adenosine-containing ligands with varying affinity and effect: ATP, ADP and AMP (Scott et al., 2004[132]). AMP binding to the γ-subunit leads the autoinhibitory part of the AMPK scaffold to detach from the KD and to bind to the γ-subunit instead, increasing the chances of phosphorylation of AMPK (Hardie et al., 2016[56]; Yan et al., 2018[164]). 

Using crystal molecule structures, it has been established that the 4^th^ CBS binding site is almost always bound to AMP, the 1^st^ and 3^rd^ binding sites competitively bind AMP, ATP and ADP while the 2^nd^ is always vacant (Hardie et al., 2016[56]; Oakhill et al., 2010[110]; Yan et al., 2018[164]). The differential adenine nucleotide binding modulates AMPK activity (Yan et al., 2018[164]).

ATP inhibits all the effects of AMP, while ADP mimics some. This means that the binding status of the three AMPK subunits acts as a censor of cellular AMP: ATP and ADP: ATP. Both these levels increase during periods of cellular stress such as exercise. Even in the presence of many-fold ATP over AMP, AMPK can sense changes in the AMP levels thanks to the complex cooperative binding of AMP in the γ-subunit. This review found that in the studies that measured AMPK levels pre and post-exercise, AMPK levels were increased (Bae-Gartz et al., 2020[9]; Ghareghani et al., 2018[41]; Moon et al., 2013[100]; Ok et al., 2018[111]). Of note, AMPK has an Akt phosphorylation site on the C terminal domain of the alpha subunit, which, when bound by Akt, modulates the AMPK/LKB1 complex increasing chances of AMPK phosphorylation (Yan et al., 2018[164]), making it a potential therapeutic target. 

Pharmacological activation of AMPK has been successful in alleviating mitochondrial dysfunction and ER stress conditions (Li et al., 2015[84]). This activation also modulates the glucose uptake in skeletal muscles and ameliorates the ATP and energy substrate handling, even in patients with neurological disorders (Browning et al., 2004[16]). 

Overall, there is indirect activation, which mainly targets the alteration of the AMP/ ADP: ATP ratio (Zhou et al., 2001[172]) or inhibition of complex I or Na/Glucose cotransporter (Hawley et al., 2016[57]). These molecules generally induce mitochondrial uncoupling resulting in a reduction of the available ATP therefore activating the AMPK. Drugs; such as metformin; activate AMPK dependent on the organic cation transporters, which are ubiquitous in the liver (Shu et al., 2007[136]). Other molecules have more affinity to the activation of AMPK in areas outside the liver (Jenkins et al., 2013[68]; Shaw et al., 2005[134]), which results in an improved metabolic profile commonly associated with NAFLD. 

Direct activators of the AMPK were also investigated. The 5-aminoimidazole-4-carboxamide ribonucleotide (AICAR), the monophosphate derivative of the cell-permeable precursor, is capable of stimulating the AMPK (Sullivan et al., 1994[142]). Nonetheless, this has resulted in cross inhibition of other AMP-sensitive enzymes (Guigas et al., 2007[46]). On the other hand, some promising findings were produced by selective activation of α complexes by 5-(5-hydroxyl-isoxazol-3-yl)-furan-2-phosphonic acid (compound 2) (Hunter et al., 2014[65]). This approach may shed the light on the scope of selective activation of such key pathway elements for NALFD reversal.

## Therapeutic Targeting of AMPK Pathway in NAFLD

**Figure 4:**
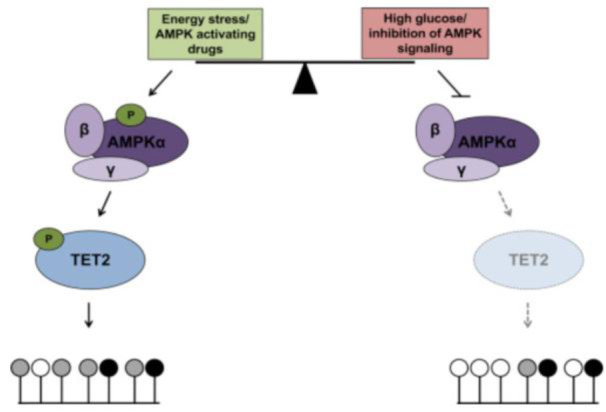
Pharmacological interference or general energy stress of AMPK pathway on epigenetic levels as described by (Wu et al., 2018).

The experimental study revealed that liver-specific AMPK knockout worsens liver lipid accumulation, steatosis, fibrosis, and inflammation, with a notable increase in apoptotic hepatocytes in AMPK knockout mice. These findings highlight AMPK's potential as a target for preventing and treating NAFLD. Recently, Zhu and colleagues revisited clinical trials targeting NAFLD (Zhu et al., 2024[173]), where AMPK activator (Metformin) was in clinical trial phase III. Lee and co-workers identified the AMPK-ULK1 axis as crucial for protecting against lipotoxicity through an atypical KEAP1-NFe2L2 pathway dependent on SQSTM1. By enhancing the interaction between AMPK and ULK1, SQSTM1 promotes ULK1 phosphorylation, induces autophagy in response to fatty liver toxicity, and activates the Keap1-Nrf2 signaling pathway, offering protection from lipotoxicity (Lee et al., 2020[83]). The pathophysiology of NAFLD is complex, with insulin resistance (IR) being the most significant factor involved throughout the entire process. Consequently, reversing IR is critical for the treatment of NAFLD. AMPK agonists have been shown to enhance IR by promoting liver lipid synthesis, increasing fatty acid oxidation, and repairing mitochondrial function. Additionally, numerous studies suggest that regulating adipocytokine production or the expression of adipocyte-specific genes is one of the most effective strategies to improve IR. For instance, adiponectin, a cytokine secreted by adipocytes, is particularly associated with IR and plays a role in regulating liver lipid metabolism. Adiponectin enhances insulin sensitivity by binding to its receptors, AdipoR1 and AdipoR2. Moreover, it can activate AMPK activity, further improving insulin signal transduction in adipose tissues, reversing IR, and preventing the onset and progression of NAFLD (Wang et al., 2019[158]). Epigenetic changes contribute to the complex transcriptional responses associated with WAT lipolysis, hepatic de novo lipogenesis, and hepatic gluconeogenesis. While these metabolic responses may hypothetically be altered with acute and chronic exercise. However, direct testing is lacking (Axsom et al., 2021[8]). Thus, targeting AMPK may represent a promising approach for the prevention and treatment of NAFLD. Wu et al., describe a “phospho-switch” in which AMPK, when it is active due to pharmacological interference or general energy stress, phosphorylates and stabilizes TET2, the enzyme that converts 5-methylcytosine (5mC) to 5-hydroxymethylcytosine (5hmC). Under conditions that inhibit AMPK signaling, such as high glucose, AMPK is no longer phosphorylated and thus inactive, resulting in a loss of TET2 phosphorylation and stability, decreased TET2 levels, and reduced 5hmC levels. This novel signaling pathway defines a mechanism by which the metabolic state of a cell can affect its epigenome, and thus gene expression in a potentially stable and heritable manner, in response to the cellular environment (Wu et al., 2018[160]) (Figure 4(Fig. 4)).

## Conclusion and Limitations

This review summarized experimental evidence of improvements in NAFLD with exercise in the absence of weight loss. Exercise ameliorated all processes known to contribute to NAFLD including, but not limited to, decreasing de novo lipogenesis, inflammation, apoptosis, and oxidative stress, whilst increasing fatty acid oxidation and autophagy. Several compounds were shown as potential pharmacological targets for inducing the observed exercise effect on NAFLD. The pathways involved appeared to have a common denominator: AMPK. Due to limitations in animal models featuring NAFLD/NASH (Hammad 2013[51]; Teufel et al., 2016[147]), there is a need to explore the potential of AMPK-targeted treatment for NAFLD to counter the pandemic of NAFLD and its complications. 

## Outlook

While the existing body of evidence highlights the significant role of exercise in mitigating the progression of NAFLD, there remain critical gaps in knowledge that future research must be addressed. One key area is the need for optimized and standardized exercise regimens tailored to specific patient populations, such as individuals with varying stages of NAFLD, age groups, or comorbid conditions. Longitudinal studies incorporating a combination of aerobic, resistance, and high-intensity interval training (HIIT) are essential to determine the most effective and sustainable exercise interventions for clinical outcomes. The interplay between exercise and other lifestyle modifications, including dietary interventions, requires deeper exploration. Unraveling the synergistic mechanisms of these combined approaches will aid in formulating comprehensive therapeutic strategies. Additionally, personalized exercise prescriptions based on genetic, metabolic, and microbiome profiles hold promise but necessitate further investigation through precision medicine frameworks. On the molecular front, emerging biomarkers of exercise-induced hepatic improvement should be validated in large-scale human cohorts. This would facilitate early identification of responders to exercise-based therapies and enable real-time monitoring of treatment efficacy. Moreover, leveraging advanced imaging techniques and multi-omics technologies could enhance our understanding of the intricate pathways linking exercise to NAFLD amelioration. Finally, translating research findings into public health initiatives is imperative. Developing accessible exercise programs, integrating behavioral counseling, and employing digital health technologies such as wearables and mobile applications could increase adherence and engagement. Policymakers must also prioritize funding and infrastructure to support community-level interventions targeting NAFLD prevention and management through physical activity. By bridging these gaps, we can advance the role of exercise from an ancillary to a cornerstone therapy in the fight against NAFLD and its associated metabolic disorders.

## Notes

Mohamed Bekheit and Seddik Hammad (Molecular Hepatology Section, Department of Medicine II, Medical Faculty Mannheim, Heidelberg University, Theodor-Kutzer-Ufer 1-3, 68167, Mannheim, Germany; E-mail: seddik.hammad@medma.uni-heidelberg.de) contributed equally as corresponding author.

## Declaration

### Funding

This study was supported by the BMBF (German Federal Ministry of Education and Research) Project LiSyM (Grant PTJ-FKZ: 031L0043), LiSyM- Cancer (Grant PTJ-FKZ: 031L0257A and 031L0314A), and the Stiftung für Biomedizinische Alkoholforschung and funding for SH. 

### Conflict of interest

None.

## Figures and Tables

**Table 1:**
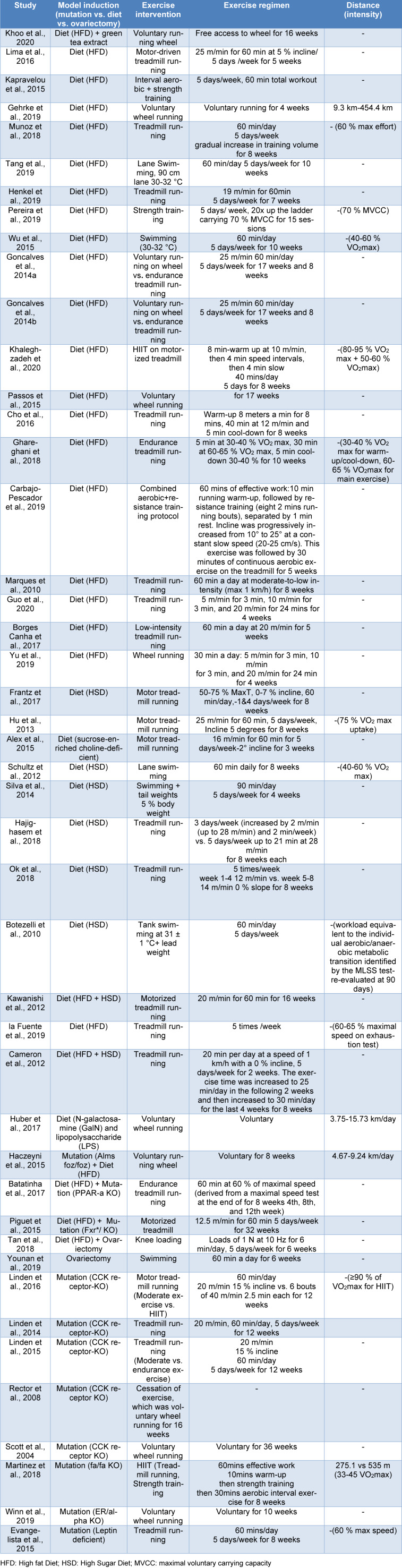
The studies including the exercise interventions are summarized

**Figure 1:**
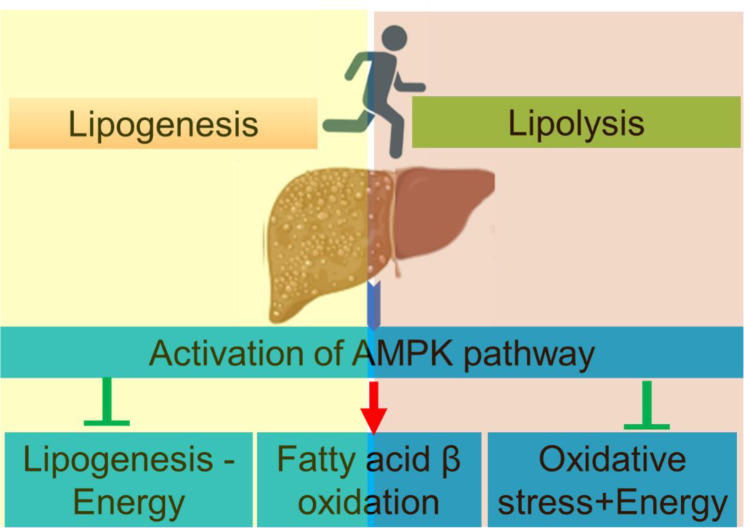
Graphical abstract: This graphical abstract illustrates how exercise impacts cellular and molecular mechanisms in non-alcoholic fatty liver disease (NAFLD) via AMPK activation. Exercise stimulates adiponectin release, influencing macrophages and intracellular pathways, which converge on AMPK. Activated AMPK enhances mitochondrial fatty acid β-oxidation, reduces lipogenesis, oxidative stress, apoptosis, and inflammation, and promotes autophagy while inhibiting SREBP1c, collectively mitigating NAFLD progression.
